# A Method to Deliver Automated and Tailored Intervention Content: 24-month Clinical Trial

**DOI:** 10.2196/38262

**Published:** 2022-09-06

**Authors:** Hailey N Miller, Corrine I Voils, Kate A Cronin, Elizabeth Jeanes, Jeffrey Hawley, Laura S Porter, Rachel R Adler, Whitney Sharp, Samantha Pabich, Kara L Gavin, Megan A Lewis, Heather M Johnson, William S Yancy Jr, Kristen E Gray, Ryan J Shaw

**Affiliations:** 1 School of Nursing Duke University North Carolina, NC United States; 2 Duke Global Digital Health Science Center Duke University Durham, NC United States; 3 Department of Surgery School of Medicine & Public Health University of Wisconsin-Madison Madison, WI United States; 4 William S Middleton Memorial Veterans Hospital Madison, WI United States; 5 Duke Office of Clinical Research School of Medicine Duke University Durham, NC United States; 6 Department of Psychiatry & Behavioral Sciences School of Medicine Duke University Durham, NC United States; 7 Center for Surgery and Public Health Brigham and Women’s Hospital Boston, MA United States; 8 Department of Medicine School of Medicine & Public Health University of Wisconsin-Madison Madison, WI United States; 9 RTI International Research Triangle Park, NC United States; 10 Division of Cardiology Charles E. Schmidt College of Medicine Florida Atlantic University Boca Raton, FL United States; 11 Christine E Lynn Women's Health & Wellness Institute/Baptist Health South Florida Boca Raton, FL United States; 12 Department of Medicine Duke University School of Medicine Durham, NC United States; 13 Health Services Research & Development Department of Veterans Affairs Puget Sound Health Care System Seattle, WA United States

**Keywords:** text message, weight management, automation, clinical trial, engagement, digital technology, electronic data capture, REDCap, automated text message, digital health intervention, health intervention, health database, digital health

## Abstract

**Background:**

The use of digital technologies and software allows for new opportunities to communicate and engage with research participants over time. When software is coupled with automation, we can engage with research participants in a reliable and affordable manner. Research Electronic Data Capture (REDCap), a browser-based software, has the capability to send automated text messages. This feature can be used to automate delivery of tailored intervention content to research participants in interventions, offering the potential to reduce costs and improve accessibility and scalability.

**Objective:**

This study aimed to describe the development and use of 2 REDCap databases to deliver automated intervention content and communication to index participants and their partners (dyads) in a 2-arm, 24-month weight management trial, Partner2Lose.

**Methods:**

Partner2Lose randomized individuals with overweight or obesity and cohabitating with a partner to a weight management intervention alone or with their partner. Two databases were developed to correspond to 2 study phases: one for weight loss initiation and one for weight loss maintenance and reminders. The weight loss initiation database was programmed to send participants (in both arms) and their partners (partner-assisted arm) tailored text messages during months 1-6 of the intervention to reinforce class content and support goal achievement. The weight maintenance and reminder database was programmed to send maintenance-related text messages to each participant (both arms) and their partners (partner-assisted arm) during months 7-18. It was also programmed to send text messages to all participants and partners over the course of the 24-month trial to remind them of group classes, dietary recall and physical activity tracking for assessments, and measurement visits. All text messages were delivered via Twilio and were unidirectional.

**Results:**

Five cohorts, comprising 231 couples, were consented and randomized in the Partner2Lose trial. The databases will send 53,518 automated, tailored text messages during the trial, significantly reducing the need for staff to send and manage intervention content over 24 months. The cost of text messaging will be approximately US $450. Thus far, there is a 0.004% known error rate in text message delivery.

**Conclusions:**

Our trial automated the delivery of tailored intervention content and communication using REDCap. The approach described provides a framework that can be used in future behavioral health interventions to create an accessible, reliable, and affordable method for intervention delivery and engagement that requires minimal trial-specific resources and personnel time.

**Trial Registration:**

ClinicalTrials.gov NCT03801174; https://clinicaltrials.gov/ct2/show/NCT03801174?term=NCT03801174

## Introduction

The use of digital technologies, such as smartphones, allows for new opportunities to consistently communicate and engage with research participants. With 85% of the adult US population now owning a smartphone [1], researchers can regularly send and receive data from participants in their everyday environment. This is particularly beneficial for researchers conducting behavioral health trials, as mobile technologies facilitate reaching participants in their day-to-day environments. This allows for cues to action or requests for information to reach participants when a specific behavior change is needed to achieve the desired outcome [2]. Further, when software are coupled with automation, researchers can engage with participants over a long period of time in an efficient and affordable manner [3,4]. This creates the opportunity for interventions to be scaled and reach populations across geographies.

The use of automated software to augment intervention content delivery offers several benefits. First, it can drastically reduce the need for personnel time. Using study staff to manually deliver intervention content to participants requires consistent staff effort. However, if software are leveraged, automated intervention content delivery can often reduce the amount of personnel time needed after initial programming [5]. Further, software can allow for intervention content to be tailored, which has shown to be an effective strategy for behavioral interventions [6,7]. The timing of delivery and information requests can be tailored to characteristics of the individual or population, such as an individual’s behavior change goals, daily habits, or stage of behavior change, and in doing so, making the intervention content more relevant to the recipient [2]. For example, the frequency of contact can be programmed to be greater during the weight loss initiation stage and lower during the weight loss maintenance stage [8]. Similarly, the messaging content can be programmed to be different dependent on individualized goals, such as eating more fruits and vegetables or walking daily. This engagement can be expanded to support persons as well. Moreover, because software can maintain a record of the message delivery schedule, fidelity increases with automation. Thus, developing affordable and widely accessible strategies to communicate and engage with participants over time can improve intervention delivery.

Research Electronic Data Capture (REDCap), a browser-based software developed by Vanderbilt University for clinical research data collection and management, is increasingly available to academic institutions across the United States and in other countries, with over 5900 institutional partners in 145 countries [9,10]. REDCap has the capability to send automated text messages. Previous literature has described REDCap’s ability to send automated communication, specifically in the context of automated medication reminders [11], symptom monitoring [12], postprocedure communication [13,14], and data collection surveys via text message over time [15]. It also has been previously used to support intervention workflow and content delivery [16-18]. However, the current literature does not include an in-depth report of REDCap being used to automate tailored intervention content and communication to research participants in a behavioral health clinical trial with longitudinal outcomes. Further, we are not aware of literature describing this approach to impart common or differentiated information between and within dyads. Given REDCap’s broad use amongst academic institutions, and its affordability to research teams at institutions with licenses, it is an ideal platform to use for automating intervention communication in this context.

In this methods paper, we present a case study and lessons learned in which we use REDCap to deliver automated text messages to participants and their partners (dyads) in a weight management trial, Partner2Lose [19]. Our goal was to create an affordable, accessible method to communicate with research participants over time.

## Methods

### Study Design and Overview

Details of the Partner2Lose protocol have been published [19]. In brief, Partner2Lose was a parallel, 2-arm randomized controlled trial that carried out a comparative evaluation of a partner-assisted intervention and a participant-only intervention for weight management. The primary outcome was participant weight at 24 months. To be eligible to enroll, participants must have (1) been cohabitating with a partner, (2) had a BMI of 27-29.9 kg/m^2^ and one obesity-related comorbidity or a BMI of ≥30 kg/m^2^, and (3) had a desire to lose weight. Partners had to have a BMI ≥18.5 kg/m^2^ to participate. Additional inclusion and exclusion criteria can be found in the previously published protocol.

Enrollment for Partner2Lose started in January 2019. Five cohorts of 45-50 couples were sequentially recruited and randomized to the participant-only arm or partner-assisted arm. The intervention included 6 months of weight loss initiation and 12 months of weight maintenance, followed by a 6-month period of no intervention. In the partner-assisted arm, partners participated in the intervention alongside the index participant and received communication skills training. Data collection will be completed in March 2023.

Index participants in both study arms received the nutrition, physical activity, and weight management intervention, a standard reduced-calorie weight management approach established in previous trials [20,21]. The weight loss initiation phase consisted of group classes every 2 weeks co-led by a registered dietitian and exercise physiologist and focused on unique dietary and physical activity education topics. At each class, participants were asked to select a goal topic from a menu of 3-4 of them (see examples in Table 1) related to the class education topics. Participants provided their goal selection to a research staff member after the group meeting. These goal selections were used to inform tailored text messages to participants.

Partners in the partner-assisted arm also received communication and support skills training [22,23]. Partners attended half of the group meetings, where they received the same nutrition and activity education as index participants. The participants and their partners were asked to select a partner support plan to support the participants’ goal from a prespecified list of options (see Table 1). Partners provided their support plan selection to a research staff member after the group meeting. These support plans were used to inform tailored text messages to partners.

During month 7, participants transitioned to the weight loss maintenance phase, where participants started to receive telephone support from the registered dietitian. During each of the 8 calls that were delivered, participants reflected on satisfaction with outcomes of weight loss and formed relapse prevention, self-monitoring, and social support plans. Partners in the partner-assisted joined 5 telephone calls during this period.

**Table 1:** Class schedule and associated goal selections for the Partner2Lose weight loss initiation phase.

Class schedule	Associated goals for participants	Associated support plan for partners
Class 1: Introduction to a low-calorie diet	—^a^	—
Class 2: Interpreting food labels and setting SMART (specific, measurable, attainable, relevant, and timebound) goals	Serving sizes Meals and snacks Calorie meal plan Fiber	Do it together Provide gentle reminders Praise your partner Remember the long game Check-in with your partner Be mindful of how your choices affect your partner’s goals Talk with your partner to develop a support plan at home
Class 3: Tracking diet and activity	Planning Measuring Tracking	Do it together Provide gentle reminders Praise your partner Remember the long game Check in with your partner Be mindful of how your choices affect your partner’s goals Talk with your partner to develop a support plan at home
Class 4: Grocery shopping	Shopping list Healthy snacks Whole grains Produce	Do it together Provide gentle reminders Praise your partner Remember the long game Check in with your partner Be mindful of how your choices affect your partner’s goals Talk with your partner to develop a support plan at home
Class 5: Meal planning	Meals Snacks Grocery lists Recipes	Do it together Provide gentle reminders Praise your partner Remember the long game Check in with your partner Be mindful of how your choices affect your partner’s goals Talk with your partner to develop a support plan at home
Class 6: Healthy cooking and modifying recipes	Dairy Meat Preparation Healthy recipes	Do it together Provide gentle reminders Praise your partner Remember the long game Check in with your partner Be mindful of how your choices affect your partner’s goals Talk with your partner to develop a support plan at home
Class 7: Dining out	Restaurant menus Modifications Planning Meals for home	Do it together Provide gentle reminders Praise your partner Remember the long game Check in with your partner Be mindful of how your choices affect your partner’s goals Talk with your partner to develop a support plan at home
Class 8: Dining out advanced practice	Substitutions Portion size Temptations On the side	Do it together Provide gentle reminders Praise your partner Remember the long game Check in with your partner Be mindful of how your choices affect your partner’s goals Talk with your partner to develop a support plan at home
Class 9: Physical activity	Planning Daily activity Try something new	Do it together Provide gentle reminders Praise your partner Remember the long game Check in with your partner Be mindful of how your choices affect your partner’s goals Talk with your partner to develop a support plan at home
Class 10: Eating more fruits and vegetables	Meals Snacks New produce Preparation	Do it together Provide gentle reminders Praise your partner Remember the long game Check-in with your partner Be mindful of how your choices affect your partner’s goals Talk with your partner to develop a support plan at home
Class 11: Mindful eating	Distractions Triggers Strategies Types of mindless eating	Do it together Provide gentle reminders Praise your partner Remember the long game Check in with your partner Be mindful of how your choices affect your partner’s goals Talk with your partner to develop a support plan at home
Class 12: Emotional eating	Emotions Triggers High-risk foods Coping strategies	Do it together Provide gentle reminders Praise your partner Remember the long game Check in with your partner Be mindful of how your choices affect your partner’s goals Talk with your partner to develop a support plan at home

^a^None determined.

### Software Used to Schedule and Deliver Text Messages

To maximize the opportunity for scalability, it is beneficial to automate components of the intervention where appropriate. To that end, we used REDCap to automate the delivery of text message communications to participants and their partners for the duration of the intervention. Specifically, 2 databases were developed, which ran in parallel: a weight loss initiation database and a weight maintenance and reminder database. The choice to develop 2 databases was to simplify the database building processes, as the databases were used for different purposes and thus had different programming requirements (described below).

The weight loss initiation database was used to reinforce class contents and support goal achievement during the weight loss initiation phase (months 1-6). The weight maintenance and reminder database was used to deliver a battery of automated reminders throughout the 24-month study period. It was also used to deliver automated text messages that concentrated on behavioral maintenance principles (eg, relapse prevention) during the weight maintenance phase (months 7-18). Both REDCap databases stored text message contents and participant-level information, including role (ie, participant or partner), record ID, and cell phone number.

**Figure 1:**
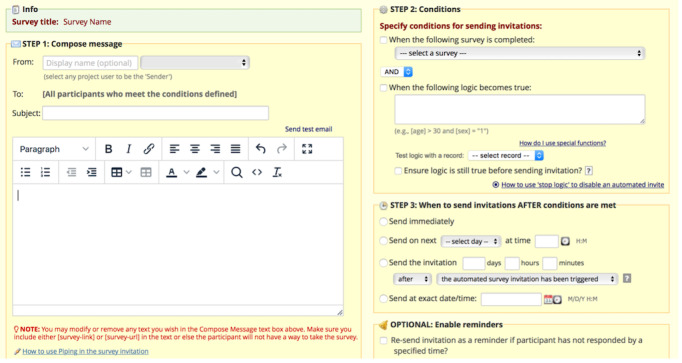
Automated Survey Invitations in REDCap used to send text messages related to goals in Partner2Lose.

To specify text message content, conditions, and schedule, we used a feature in REDCap, known as “Automated Survey Invitations” (Figure 1). This feature allows messages to be sent, via email or text message, at a designated time (eg, immediately, in 2 hours, or in 14 days) after a set of prespecified conditions are met. Conditions are specified from data fields in the respective REDCap database, such as participant role. An example condition for the goal database would be “[participant role=partner].” Under this condition, the messages would only be sent to someone if their documented role in REDCap was “partner.” Conditions used for the databases are described in greater detail below.

The default for the Automated Survey Invitation feature in REDCap is for the messages to include a hyperlink to a survey for the recipient to complete. For this study, however, text messages were unidirectional and were not intended to solicit a response; hence, we did not want to send a hyperlink to a survey in our text message. Therefore, we used an open HTML comment to eliminate the hyperlinked survey feature in the body of the Automated Survey Invitation to make the system deliver a plain text message.

To deliver the text messages to participants, we used a third-party communication application integrated within REDCap, known as Twilio (Twilio Inc). Twilio is a cloud communication platform that uses web services application programming interfaces (APIs) to make and receive phone calls and text messages. To do so, a Twilio account, 34-digit string identifier (SID), authentication token (Auth Token), and purchased phone number are required. One Twilio phone number can be used for the duration of the study and for all participants. The Account SID and Auth Token act as the Twilio account’s username and password and are used to inform Twilio from which account the API requests are derived. As such, we created a Twilio account to retrieve an Account SID and Auth Token, which we entered into our REDCap databases. We disabled the “Request Inspector” feature on our Twilio account to ensure that the server did not store participant information. Disabling this feature is a requirement by our institution to remain HIPAA (Health Insurance Portability and Accountability Act)-compliant.

### Development of Text Message Conditions

#### Weight Loss Initiation Database: Automated and Tailored Biweekly Messaging From Baseline to 6 Months

**Figure 2:**
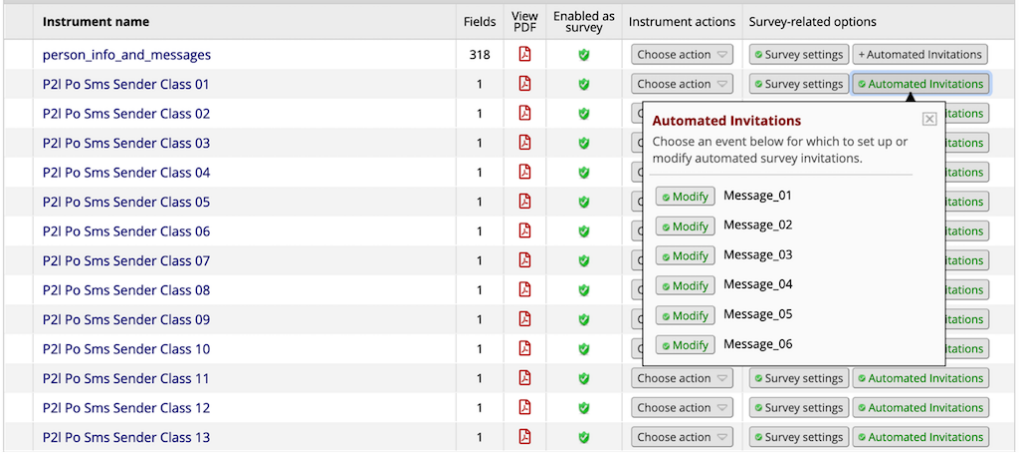
Partner2Lose weight loss initiation database.

The weight loss initiation database was programmed to send participants (both arms) and their partners (partner-assisted arm) tailored text messages in 2-week increments during the first 6 months of the intervention to reinforce class content and support goal achievement. As described previously, participants and their partners were prompted to choose a goal and support plan, respectively, following each group class and to provide their selections to a study team member. Participants’ selections, along with which class they attended (class number), were entered into REDCap by a study team member. This documentation triggered a 2-week battery of automated messages on a standard delay of 2, 4, 6, 8, 10, and 12 days (see example of the database in Figure 2). The participant’s role and class number were used as conditions to tailor the 2-week battery of messages for each participant (eg, [participant role=partner] AND [class number=class 5]). The goal and support plan selections were piped into text messages, when relevant, to individualize text message content (see Table 2 for example).

**Table 2:** Text message algorithm example for class 2: interpreting food labels and setting SMART (specific, measurable, attainable, relevant, and timebound) goals.

Participant role	Class schedule	Goal selection	Text message battery
Participant in participant-only arm	Class 2: Interpreting food labels and setting SMART goals	Participant goal: meals and snacks Partner goal: not applicable	Day 2: Your goal for the next two weeks focuses on: Meals and snacks [populated from goal chosen in Table 1 from a menu of 3-4 options] Day 4: Remember to look at the nutrition facts label and stick to 1 serving to stay within your calorie budget Day 6: Keep an eye on added sugars Day 8: Remember to work on the goal you set for: Meals and snacks [populated from goal chosen in Table 1 from a menu of 3-4 options] Day 10: How much fiber have you been consuming? Fiber helps us stay full longer. Try for at least 25g/day Day 12: Try comparing the serving sizes of food using volume (cup measures) vs. weight (food scale). How do they compare?
Participant in partner-assisted arm	Class 2: Interpreting food labels and setting SMART goals	Participant goal: meals and snacks Partner goal: praise your partner	Day 2: Text 1: Your goal for the next two weeks focuses on: Meals and snacks. Text 2: Your partner's support plan is: Praise your partner Day 4: Support tip: When trying to problem-solve together: state the issue, say why it's important, discuss possible solutions, and try a solution. Day 6: Keep an eye on added sugars. Day 8: Text 1: Check in with your partner about whether their support plan is helping you reach the goal you set for: Meals and snacks. Text 2: Check in with your partner about how their support plan is going, which is: Praise your partner Day 10: Support tip: When making decisions with your partner, remember to talk about what each person needs out of the solution. Day 12: Try comparing the serving sizes of food using volume (cup measures) vs. weight (food scale). How do they compare?
Partner in partner-assisted arm	Class 2: Interpreting food labels and setting SMART goals	Participant goal: meals and snacks Partner goal: praise your partner	Day 2: Text 1: Your partner's goal for the next two weeks focuses on: Meals and Snacks. Text 2: Your support plan is: Praise your partner Day 4: Support tip: When trying to problem-solve together: state the issue, say why it's important, discuss possible solutions, and try a solution Day 6: Keep an eye on added sugars Day 8: Text 1: Check in with your partner about their goal, which focuses on: Meals and Snacks. Text 2: Check in with your partner about how you are doing with your support plan, which is: Praise your partner Day 10: Support tip: When making decisions with your partner, remember to talk about what each person needs out of the solution Day 12: Try comparing the serving sizes of food using volume (cup measures) vs. weight (food scale). How do they compare?

#### Maintenance and Reminder Database: Automated Messaging From Baseline to 24 Months

The weight maintenance and reminder database was programmed to send a total of 80 automated text messages to each participant (both arms) and 70 automated text messages to their partners (partner-assisted arm) over the 24-month study period. These text messages were used to remind participants and their partners of group classes, dietary recall and physical activity tracking for assessments, and measurement visits. They were also used to reinforce behavior maintenance principles during the weight loss maintenance phase of the study (months 7-18). A REDCap field was created for each possible text message at baseline. In each field, we specified the text message conditions and delivery schedule and piped in the corresponding text message content. Then, we set conditions to specify which participants should receive the text message. Conditions used in the 2-year database were the role and number of days since baseline (eg, [participant role=partner] AND [days since baseline=80]). To customize delivery dates for each cohort, we used an external date calculation module (date calculation fields, version 1.64) in REDCap. This individualization took into consideration the schedule of group meetings.

### Cost of Text Messaging

Twilio has a pay-as-you-go payment structure and does not require an upfront fee to make an account. A monthly fee of US $1.00 for a Twilio phone number is required. Phone numbers can be selected on the basis of a local area code. At the time of this intervention, text messaging to domestic numbers costs US $0.0075 per text message.

### Intervention Fidelity

**Figure 3:**
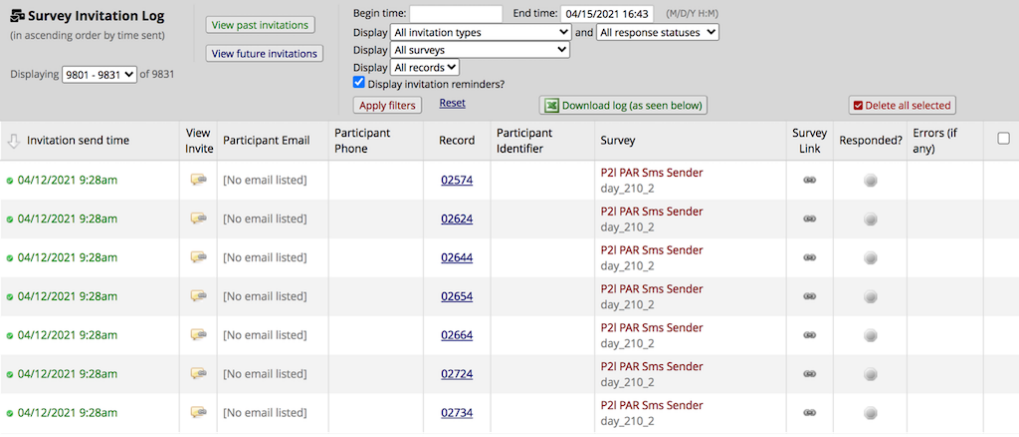
Partner2Lose text message delivery log in Research Electronic Data Capture (REDCap).

REDCap automatically maintains a log display or comprehensive list of messages that are delivered or scheduled to be delivered (Figure 3). The log displays the date and time when the message was sent or is scheduled to be sent, as well as the participant’s cell phone number and record ID. The log also displays if there were any errors. The log can be viewed and downloaded at any time to monitor intervention fidelity. Intervention fidelity is further enhanced over long periods of time through automation and limiting the need for a person to send a message. Our study team looked at the log monthly to check for errors and confirm message delivery. If an error was seen, we investigated and resolved it.

### Ethics Approval

Approval for Partner2Lose was obtained by the University of Wisconsin Health Sciences Institutional Review Board in December 2018 (protocol 2018-1400).

## Results

A total of 1061 participants and partner dyads were screened to participate in Partner2Lose. Among these dyads, 3 participants were excluded from the study for not having a smartphone with a data or texting plan. No partners were excluded for this reason. Five cohorts comprising 231 couples were consented and randomized in the Partner2Lose trial. The initial build of the 2 databases took approximately 120 hours. Thereafter, the databases have required, on average, less than 15 minutes of maintenance each week.

As of February 22, 2022, the initiation and maintenance and reminder databases have sent 22,887 and 21,447 Automated Survey Invitations via text message, respectively. A total of 53,518 text messages are planned to be delivered by study completion (March 2023).

The cost to send these text messages via Twilio will be approximately US $450 for the full study duration. Of the 44,334 text messages that have been sent, 217 failed to deliver, equating to a 0.004% error rate. These errors appear to occur at random and consistently over time. REDCap did not provide more information regarding the messages that failed to deliver, which could indicate error on the participants’ side, which we were unable to identify. In one instance, though, study staff reported that participants said they received multiple repeated messages. This was a bug found within the REDCap logic, and we were able to quickly address the problem. Additional errors have not been noted within REDCap or from study participants thus far.

## Discussion

### Principal Findings

To effectively implement interventions in large populations, scalable intervention delivery approaches are needed. This study demonstrates a feasible approach to automate a significant component of participant communication and engagement over a 24-month behavioral weight management trial using a widely available software, REDCap.

The widespread use of mobile technology has expanded our opportunity to automate the delivery of intervention content without requiring face-to-face interactions and with minimal upkeep and maintenance by study staff [1]. Inasmuch, it reduces study burden and limits the need to travel to a study site. This is particularly useful for populations in rural areas or those who may work multiple jobs or have caregiving responsibilities. Text messaging is also cost effective, as indicated in our report and previous research [24], and unlike other digital interventions and strategies, it does not require a smartphone (with access to applications) or other devices, such as wearables, facilitating reach to sociodemographically diverse groups [1,25]. In fact, 97% of adults own a cellphone in the United States, with high rates across all race and ethnicity, household income, and educational attainment groups [1].

There are other examples of automated, tailored interventions being delivered in behavioral health trials. These interventions have used other software tools that require individual development and may involve smartphone apps. One example is the Nourish study [26] that leverages a smartphone application and evidence-based behavior change principles to improve adherence to the DASH eating pattern among adults with hypertension. The Nourish intervention delivers tailored and personalized feedback that is based on dietary tracking and goal attainment. This feedback also takes into consideration personal characteristics of the participant, such as working full time, to inform the feedback provided. Given the increased number of variables being considered in the feedback algorithm, in addition to it relying on an external application, REDCap likely would not be a feasible option for this study or others alike. These are important factors to consider when selecting a software platform. We chose REDCap for this study owing to institutional investment in which it supports a wide variety of research needs, and REDCap’s wide adoption and collaborative, international network. We also only sought to leverage text messaging for intervention delivery owing to its simplicity and equity in use across diverse populations. Thus, results from this study can be more easily replicated.

The REDCap Consortium is an international community of software platform partners [10]. While each institution maintains its own version of the software, developed features are shared across the community. This allows for interoperability of data transfer across sites and best practices related to security and regulatory requirements. The initial costs of using software such as REDCap requires institutional investment; however, there is less of a need to rebuild custom software tools across the enterprise once the infrastructure exists, resulting in reduced long-term spending [27]. Using REDCap, or a similar platform, is also cost savings for individual study teams, as the scalability of automated processes is exponential and more affordable than manual processes that incur labor costs over time. For example, increasing a sample size from 100 to 10,000 would traditionally require a significant increase in labor costs and data tracking; however, software and automation can greatly reduce the per-participant cost.

Automating the participant communication component of our intervention delivery increased our ability to monitor and manage intervention fidelity. Specifically, using REDCap’s survey invitation log feature, we were able to identify messages delivered or scheduled to be delivered that had an error. This feature allowed us to make informed and responsive modifications such as reinitiating messages or stopping double messaging. It should be noted that our intervention did not ask or prompt participants to respond to text messages, so there could be delivery errors not identified by REDCap or our team. Nonetheless, the databases used in this study demonstrated a high rate of fidelity over time in message delivery as demonstrated by the very low error rate.

The tools and features in REDCap are regularly modified and updated to improve the platform’s functionality and effectiveness. As such, the workflow described in this paper would benefit from being adapted to leverage the tools and features available today. For example, if we were to implement automated text messaging in our next trial, we would use REDCap’s “Alerts and Notifications” feature to send the text messages via Twilio. This relatively newer feature provides an option to specify delivery conditions to tailor the text message content by each participant, however, does not require the text message to be connected to a survey in REDCap. The feature described to send text messages in this report, Automated Survey Invitations, would be more appropriate to send text messages or communications that solicit a survey response, such as an outcome survey or adverse event questionnaire. Nonetheless, both of these features have tracking logs, in which intervention delivery and survey completion can be tracked and maintained by the study team.

### Limitations

We note several limitations to automating intervention content and communication using REDCap and Twilio, which should be considered before design and implementation. First, it requires participants to have a consistent mobile phone, which may limit the generalizability of the automated communication approach. Although mobile phone ownership is high and continues to grow in the United States [1], there is still a small percentage of individuals who do not own a mobile phone and thus cannot be reached using this approach. For example, 8% of adults aged 65 years or older do not own mobile phone and would be inaccessible via this modality [1]. Second, the phone number that is used to deliver the text messages via Twilio is a robot. Thus, if someone were to reply to the text message, there would not be a study team member monitoring the responses or be available to reply. This can be a safety concern if a participant is seeking medical advice from a study team member; however, to minimize this concern, we notified participants at the start of the trial that responses would not be monitored. Additionally, it is worth noting that participants received messages from the same phone number over the course of the study and were able to save the number so they would not think it was spam. Twilio has the capability for 2-way SMS text messaging; however, at the time of implementation, REDCap only had the capability of receiving text messages that met predefined criteria. For safety or technical concerns, we provided alternative contact modalities for the participants to reach the trial staff, including an email ID and a phone number. Third, although our data indicate that the error rate was small in this study, it is possible that errors went unidentified as our intervention did not prompt a response from participants. Moreover, if there is a programming error, it is likely to impact several study participants rather than a single participant. Fourth, given the use of text messaging, it was not possible to record if a participant read the intervention communication. Lastly, accounting for holidays and weather can be challenging, and automated messaging may occur when not desired by participants. In the future, this may be mitigated by asking participants communication preferences or tailoring text messages to times of the day that are best suited to them.

### Conclusions

The proliferation of mobile phones coupled with research management software offers study teams the opportunity to integrate automation at scale into their intervention implementation and delivery. Our trial automated the delivery of intervention content and communications using REDCap for 5 cohorts of participants and their partners in a behavioral health trial with longitudinal outcomes. Our approach provides a framework that can be used in future behavioral health interventions to create an affordable, reliable and accessible method for intervention engagement.

## Abbreviations

API: application programming interface
Auth: authentication
HIPAA: Health Insurance Portability and Accountability Act
REDCap: Research Electronic Data Capture
SID: string identifier
SMART: specific, measurable, attainable, relevant, and timebound
